# Biological functions of hCG and hCG-related molecules

**DOI:** 10.1186/1477-7827-8-102

**Published:** 2010-08-24

**Authors:** Laurence A Cole

**Affiliations:** 1 USA hCG Reference Service, University of New Mexico, Albuquerque NM 87131, USA

## Abstract

**Background:**

hCG is a term referring to 4 independent molecules, each produced by separate cells and each having completely separate functions. These are hCG produced by villous syncytiotrophoblast cells, hyperglycosylated hCG produced by cytotrophoblast cells, free beta-subunit made by multiple primary non-trophoblastic malignancies, and pituitary hCG made by the gonadotrope cells of the anterior pituitary.

**Results and discussion:**

hCG has numerous functions. hCG promotes progesterone production by corpus luteal cells; promotes angiogenesis in uterine vasculature; promoted the fusion of cytotrophoblast cell and differentiation to make syncytiotrophoblast cells; causes the blockage of any immune or macrophage action by mother on foreign invading placental cells; causes uterine growth parallel to fetal growth; suppresses any myometrial contractions during the course of pregnancy; causes growth and differentiation of the umbilical cord; signals the endometrium about forthcoming implantation; acts on receptor in mother's brain causing hyperemesis gravidarum, and seemingly promotes growth of fetal organs during pregnancy.

Hyperglycosylated hCG functions to promote growth of cytotrophoblast cells and invasion by these cells, as occurs in implantation of pregnancy, and growth and invasion by choriocarcinoma cells. hCG free beta-subunit is produced by numerous non-trophoblastic malignancies of different primaries. The detection of free beta-subunit in these malignancies is generally considered a sign of poor prognosis. The free beta-subunit blocks apoptosis in cancer cells and promotes the growth and malignancy of the cancer. Pituitary hCG is a sulfated variant of hCG produced at low levels during the menstrual cycle. Pituitary hCG seems to mimic luteinizing hormone actions during the menstrual cycle.

## Introduction

It is difficult to say who specifically was the discoverer of the hormone we call hCG. In 1912, Aschner stimulated the genital tract of guinea pigs with injections of a water-soluble extracts of human placenta [1]. In 1913, Fellner induced ovulation in immature rabbits with a saline extracts of human placenta [2]. In 1919, Hirose stimulated ovulation and normal luteal function in immature rabbits by repeated injection of human placental tissue [3]. All of these works show that there was a clear hormonal link between the placenta and the uterus [1-3]. In 1927, Ascheim and Zondek demonstrated that pregnant women produce a gonad-stimulating substance [4]. They showed that injecting this substance into intact immature female mice let to follicular maturation, ovulation, and hemorrhaging into the ovarian stroma. Around this time, the name human chorionic gonadotropin (hCG) was conceived: Chorion comes from latin chordata meaning afterbirth; gonadotropin because the hormone is a gonad tropic molecule, acting on the ovaries, promoting steroid production.

As we know today, hCG is a hormone comprising an α-subunit and a β-subunit which are held together by non-covalent hydrophobic and ionic interactions. The molecular weight of hCG is approximately 36,000. It is an unusual molecule in that 25-41% of the molecular weight is derived from the sugar side-chains (25-30% in regular hCG and 35-41% in hyperglycosylated hCG). Today, the function of hCG is still marked as being progesterone promotion in most medical student text books, but we now know now that hCG has numerous other important placental, uterine and fetal functions in pregnancy. From the time of implantation, hCG produced by trophoblast cells take over corpus luteal progesterone production rom luteinizing hormone (LH), acting on a joint hCG/LH receptor. This continues for approximately 3 to 4 weeks. After that time, there are sufficient syncytiotrophoblast cells in the placenta to take over progesterone production from corpus luteal cells.

Research now shows that there are at least 4 independent variants of hCG, each produced by different cells with separate biological functions. All the molecules share a common hCGβ-subunit amino acid sequence. There is hCG, produced by differentiated syncytiotrophoblast cells or more specifically villous syncytiotrophoblast cells as pregnancy progresses [5-7]. This is the molecules that promotes progesterone production by ovarian corpus luteal cells and has multiple other biological functions as described below. Hyperglycosylated hCG is a sugar variant of hCG made by root cytotrophoblast cells or extravillous cytotrophoblast cells as pregnancy progresses [6,7]. Hyperglycosylated hCG is not a hormone but is an autocrine, acting on cytotrophoblast cells to promote cell growth and invasion as in implantation of pregnancy and invasion by choriocarcinoma cells [8,9]. Free β-subunit is the alternatively glycosylated monomeric variant of hCG made by all non-trophoblastic advanced malignancies [10]. Free β-subunit promotes growth and malignancy of advanced cancers [11,12]. A fourth variant of hCG is pituitary hCG, produced during the female menstrual cycle. This molecules has sulfated rather than sialylated oligosaccharides. Pituitary hCG functions in an LH-like manner to promote follicular maturation, stigma formation and meiosis in the primary follicle, ovulation, luteinization of the follicle, and progesterone production during the menstrual cycle [13,14]. The biological activities of all 4 variants of hCG and the actions of the hCG/LH receptor are carefully investigated in this review.

The serum and urine concentration of hCG and hyperglycosylated hCG during pregnancy are investigated in this comprehensive review. The extreme variations of hCG and hyperglycosylated hCG concentration are examined and how the extreme concentrations are managed by the hCG/LH receptor are investigated. hCG binds a common receptor with LH, the LH/hCG receptor. The specificity of the receptor and mechanism of receptor action are also considered in this review.

## Biological function of hCG

The hormone hCG comprises an α-subunit and a β-subunit. The α-subunit is common to hCG, to the autocrine/paracrine hyperglycosylated hCG, to the hormone pituitary hCG, and to the hormones LH, follicle stimulating hormone (FSH), and thyroid stimulating hormone (TSH), and to the common free α-subunit formed in excess. The β-subunit of hCG, while structurally somewhat similar to the β-subunit of LH, differentiates hCG, hyperglycosylated hCG, and pituitary hCG from other molecules. Both hCG and LH bind and function through a common hCG/LH receptor. The biggest difference between LH and hCG is that LH, pI 8.0, has a circulating half-life of just 25-30 minutes [15], while hCG, pI 3.5, has a circulating half-life of approximately 37 hours [16], or 80-fold longer than that of LH. In many respects hCG is a super LH produced in pregnancy, with 80X the biological activity of LH, yet acting on the joint receptor. While LH, FSH and TSH are made by the anterior lobe of the pituitary, hCG is produced by fused and differentiated placental syncytiotrophoblast cells [6].

The original biological activity of hCG was first revealed in the nineteen twenties and confirmed and elaborated in the years that followed [1-4,17-23]. With pregnancy, hCG takes over from LH in promoting progesterone production by ovarian corpus luteal cells, preventing menstrual bleeding (Table 1). As we know today, hCG only promotes progesterone production for 3-4 weeks following pregnancy implantation. This function is active for approximately 10% of the length of pregnancy. As shown in Tables 2 and 3 hCG reaches a peak at 10 weeks of gestation, or almost one month after progesterone promotion is complete, then continues to be produced through the length of pregnancy. Clearly, progesterone production is not the principal purpose of hCG. As illustrated in Table 1, hCG has been shown in recent years to have numerous functions in the placenta, uterus and possible in the fetus during pregnancy.

**Table 1:** The biological functions of the isoforms of hCG.

Function	References
A. hCG	
1. Promotion of corpus luteal progesterone production	[1-4,17-23]
2. Angiogenesis of uterine vasculature	[24-30]
3. Cytotrophoblast differentiation	[31]
4. Immuno-suppression and blockage of phagocytosis of invading trophoblast cells	[32-38]
5. Growth of uterus in line with fetal growth	[39,40]
6. Quiescence of uterine muscle contraction	[39,41-43]
7. Promotion of growth and differentiation of fetal organs	[44-49]
8. Umbilical cord growth and development	[51-53]
9. Blastocysts signals endometrium prior to implantation	[54-56]
10. hCG in sperm and receptors found in fallopian tubes suggesting pre-pregnancy communication	[57-60]
11. hCG receptors in adult brain hippocampus, hypothalamus and brain stem, may cause pregnancy nausea and vomiting	[61,62]
12. hCG and implantation of pregnancy, hCG stimulates metalloproteinases of cytotrophoblast cell.	[64-67]
B. Hyperglycosylated hCG	
1. Stimulates implantation by invasion of cytotrophoblast cells as occurs at implantation of pregnancy, blocks apoptosis and growth and malignancy of choriocarcinoma cells.	[8,9,71,74]
2. Stimulates growth of placenta and malignant placenta by promoting growth of cytotrophoblast cells	[9,74]
C. Free β-subunit	
1. Blockage of apoptosis in no-trophoblastic malignancies, promotion of growth and malignancy	[83,85,91-95]
D. Pituitary hCG	
1. Seemingly mimics LH functions, promoting follicular growth, meiosis, stigma formation, ovulation, luteogenesis and promoting progesterone production.	[121,122]

**Table 2:** Concentration of total hCG and hyperglycosylated hCG (hCG-H) in 496 serum samples from 310 women with term pregnancies measured using the Siemens Immulite 1000 total hCG assay.

Gestation age (weeks since start of menstrual period)	N	Median Total hCG ng/ml	Range Total hCG ng/ml (variation)	Median HCG-H ng/ml	Range hCG-H ng/ml (variation)	hCG-H %
3-weeks-3-weeks 6-days	n = 42	0.26 (16 of 42 <0.1 ng/ml)	0.04 - 5.5	0.20 (16 of 42 <0.1 ng/ml)	0.01 - 6.45	87%
4 weeks-4 weeks 6-days	n = 42	3.4	0.21 - 173 (824X)	2.5	0.18 - 160 (888X)	51%
5 weeks-5-weeks 6-days	n = 67	65	1.86 - 1308 (704X)	8.6	0.96 - 698 (731X)	43%
6-weeks-6-weeks 6-days	n = 29	252	3.80 - 855 (225X)	86	0.76 - 629 (827X)	36%
7 weeks-7 weeks 6-days	n = 30	3,278	203 - 7,766 (38X)	359	27 - 931 (34X)	16%
8 weeks-8 weeks 6-days	n = 33	4,331	1,064 - 10,057 (9.4X)	386	67 - 1050 (15.6X)	7.0%
9 weeks-9 weeks 6-days	n = 24	5,832	1,031 - 11,586 (11.2X)	430	102 - 1158 (11.3X)	5.1%
10 weeks-10 weeks 6-days	n = 20	10,352	1,952 - 19,958 (10.2X)	521	188 - 1855 (9.9X)	4.3%
11 weeks-13-weeks 6-days	n = 41	5,953	1,440 - 15,318 (10.6X)	137	24 - 330 (13.7X)	2.3%
14 weeks-17 weeks 6-days	n = 57	2,934	311 - 4,757 (15.2X)	26	6.7 - 129 (19.3X)	1.3%
18 weeks-26-weeks 6-days	n = 62	1,931	210 - 6,223 (30.3X)	15.8	5.3 - 95 (17.9X)	0.65%
27 weeks-40 weeks 6-days	n = 49	1,911	184 - 8,530 (46.4X)	2.95	0.3 - 12.2 (40.6X)	0.14%

Data from 50 pregnancies that failed due to miscarriage were excluded from this table. Pregnancies which failed to implant in early pregnancy (total hCG <0.1 ng/ml) are indicated in parenthesis.

The research groups of Rao et al., Zygmunt et al., and Noel et al., have each shown that hCG also functions to promote angiogenesis and vasculogenesis in the uterine vasculature during pregnancy. This insures maximal blood supply to the invading placenta and optimal nutrition to the fetus [24-30] (Table 1). The hCG/LH receptor gene is expressed by uterine spiral arteries, and hCG acts on them to promote angiogenesis. This is probably a major function of hCG during the course of pregnancy insuring adequate blood supply or nutrition to the placenta. hCG also has an important function at the placenta trophoblast tissue level promoting the fusion of cytotrophoblast cells and their differentiation to syncytiotrophoblast cells [31,32] (Table 1). Testicular gem cell cancers take on trophoblast cytology. hCG may function similarly to promote differentiation of testicular cancer cytotrophoblast cell.

Four independent research groups showed that hCG promotes an anti-macrophage inhibitory factor or a macrophage migration inhibitory factor, a cytokine that modulates the immune response during pregnancy, which reduces macrophage phagocytosis activity at the placenta-uterine interface, preventing destruction of foreign fetoplacental tissue [33-35] (Table 1). Three other groups have shown that hCG may directly suppress any immune action against the invading foreign tissue [36-38]. All told, hCG appears to be one of the numerous factors acting to prevent rejection of the fetoplacental tissue. Most observations suggest that hCG has an inhibitory or suppressive function on macrophage activity. One group, Wan et al. [35] demonstrated that hCG can directly enhance innate immunity by stimulating macrophage function.

Multiple groups have found hCG/LH receptor in the myometrium of the uterus. It has been indicated by two groups that uterine growth in line with fetal growth may be stimulated by hCG, so that the uterus expands with fetal size during pregnancy [39,40] (Table 1). Four groups have shown that hCG relaxes myometrial contractions during the course of pregnancy. hCG acts on a BK-Ca calcium activated channel to relax to myometrium during the course of pregnancy [39,41-43]. hCG levels drop during the final weeks of pregnancy. It has been suggested that this drop may be the cause of increased contractions in the weeks prior to delivery.

Exciting new research is finding hCG/LH receptors in fetal organs. Goldsmith et al. [44], have found hCG/LH receptors in the fetal kidney and liver. Rao et al. [45-49], have located hCG/LH receptors in the lung, liver, kidneys, spleen, and small and large intestines. Interestingly, this hCG/LH receptor is present in the fetal organs but completely absent in the adult organs. It is suggested that hCG may promote organ growth and differentiation in the fetus. The human fetus might produce its own hCG from the kidneys and liver [44,50]. The concentrations of hCG in the fetal circulation, however, are much lower than maternal concentrations, suggesting that placental hCG secretion is directed towards the maternal circulation and it is prevented from entering into fetal circulation [50]. While hCG/LH receptor has been shown in fetal organs, no function has been directly demonstrated, just indicated by the presence of receptor. As such, all the findings regarding the fetus have to be considered as just suggestions at this time. Unfortunately, all animals except advanced primates do not make a form of hCG, making the role of hCG in the fetus difficult to confirm.

hCG has also been shown to function in umbilical cord growth and development [51,52]. It is interesting that hCG and hyperglycosylated hCG work together to promote the growth (growth of root cytotrophoblast cells, hyperglycosylated hCG) and differentiation (promoted by hCG) of the placenta, and promotion of the uterine blood supply to meet the invading placenta (promoted by hCG). The next step is the development of the umbilical cord and circulation. This is also seemingly promoted by hCG, suggesting hCG and hyperglycosylated hCG involvement in multiple steps of placentation and fetal development [44-49,51-53].

Multiple publications suggest a signaling occurs between the unimplanted blastocyst and the decidua tissue [54-57]. Four independent reports show that the blastocyst preimplantation secretes hCG into the uterine space which is taken up by hCG/LH receptors on the endometrial surface (Table 1). In response, the endometrium is prepared for an impending implantation [54-57]. These non-vascular communications by hCG are a critical part of successful pregnancy. Recent studies show the importance of a receptive endometrium and of hCG preimplantation signaling in a successful pregnancy [58-60]. hCG signaling directly causes immunotolerance and angiogenesis at the maternal fetal interface. hCG increases the number of uterine natural killer cells that play a key role in the establishment of pregnancy [58-60].

Other new data shows other pre-pregnancy implantation function of hCG. Publications from Rao et al. [61-63] and by Gawronska et al. [63], shows the presence of an hCG/LH receptor (shown by presence of mRNA and demonstration of receptor action) in human sperm and in the fallopian tubes (Table 1). The function of the hCG/LH receptor in sperm is unclear. It possibly has some relationship to fertility. The hCG/LH receptor in the fallopian tubes may be that which is acted on by LH, which relaxes the fallopian tube for fertilization to take place.

It has long been speculated that hCG may have a role in implantation of pregnancy [64-67]. Publications suggest an autocrine or paracrine function of hCG in implantation of pregnancy. hCG of implantation is seemingly produced by cytotropblast cells. However, hCG is an endocrine. We now know from recent research that a variant of hCG, hyperglycosylated hCG, rather than hCG itself, is produced by cytotrophoblast cells [6,7]. Hyperglycosylated hCG is an autocrine or paracrine and has been shown to directly promote implantation of pregnancy [6,8,9]. This is seeming what was considered the hCG implantation function. Hyperglycosylated hCG and its role in implantation of pregnancy are reviewed in Section 3. A recent study by Fluhr and collages [65], suggest a direct role of hCG in cytotrophoblast cell metalloproteinase production, this could be true and needs careful investigation.

Finally, the hCG/LH receptor has been demonstrated in adult women's brains. CNS receptors are present in several areas of the brain such as the hippocampus, hypothalamus and brain stem [68,69] (Table 1). The finding of an hCG receptor in these parts of the brain may explain the hyperemesis gravidarum or nausea and vomiting that occurs during normal pregnancy.

All told, hCG has a very wide range of actions through the hCG/LH receptor. hCG and hyperglycosylated hCG seemingly act to together to promote the growth and differentiation of trophoblast cells or formation of the placenta villous structures. They seemingly start their action early with blastocyst signaling of the endometrium of forthcoming implantation. Hyperglycosylated hCG then promotes implantation and growth of cytotrophoblast cells. hCG promotes the differentiation of cytotrophoblast cells to syncytiotrophoblast cells and so the villous structures which are mixture of the two cell types are formed. hCG also promotes the uterine vasculature to maximally provide blood to the hemochorial placentation structure. hCG also acts on the fetus to promote growth and differentiation of fetal organs. During this time hCG acts on the maternal brain to promote hyperemesis gravidarum. Taking everything together, hCG and hyperglycosylated hCG are the hormone and autocrine that seemingly control pregnancy.

## Biological function of hyperglycosylated hCG

Hyperglycosylated hCG is a glycosylation variant of hCG produced by root cytotrophoblast cells and extravillous cytotrophoblast cells [6,7]. It shares the amino acid sequences of the α- and β-subunit of hCG with 8 oligosaccharides side chains. While hCG has monoantennary (8 sugar residues) and biantennary (11 sugar residues) N-linked oligosaccharides, and mostly trisaccharide O-linked oligosaccharides (3 sugar residues), hyperglycosylated hCG has mostly larger fucosylated triantennary (15 sugars) N-linked oligosaccharides and double-size hexasaccharide O-linked oligosaccharides (6 sugar residues). As a result the molecular weight of hCG is 36,000, while the molecular weight of hyperglycosylated hCG is 40,000 to 41,000, dependent on extent of hyperglycosylation. The additional sugars structures on hyperglycosylated hCG seeming prevent complete folding of the αβ dimer. This exposes other receptor binding site on hyperglycosylated hCG. Common regions include a transforming growth factor beta (TGFβ)/platelet-derived growth factor (PDGF)/Nerve growth factor (NGF) common cystine-knot related structure [70]. The function of hyperglycosylated hCG, blocking apoptosis [71], and a likely metalloproteinase promoting activity [72], suggests that hyperglycosylated hCG may be an antagonist of TGFβ receptor controlled functions in cytotrophoblast cells. While these pathways seems very likely from multiple studies of placental implantation, cytotrophoblast cell apoptosis, cytotrophoblast cells and metalloproteinases and placental invasion biology, that TGFβ receptor is involved in these actions [73-86]. This still needs to be proven by needed research. Hyperglycosylated hCG appear to acts by antagonizing a cytotrophoblast TGFβ receptor, seemingly blocking apoptosis and promoting invasion by metalloproteinases [71-86].

As shown, hyperglycosylated hCG is the principal variant of hCG produced in early pregnancy. Hyperglycosylated hCG comprises an average of 87% of the total hCG produced in serum during the third week, 51% during the fourth week and 43% during the fifth week of gestation (Table 2). Hyperglycosylated hCG levels then dwindles to <1% of total hCG during the 2^nd ^and 3^rd ^trimesters of pregnancy. This is consistent with hyperglycosylated hCG having a function in promoting implantation in early pregnancy [8,9,87].

Research clearly shows that hyperglycosylated hCG acts on choriocarcinoma cells (cancer of cytotrophoblast cells) promoting invasion [9,10]. Hyperglycosylated hCG is the principal variant of hCG made by choriocarcinoma cells [9,10]. The role of hyperglycosylated hCG in choriocarcinoma invasion has been demonstrated now by 3 independent groups, each showing that this molecules promotes invasion by choriocarcinoma cells in Matrigel chambers [9,71,88]. Other studies examine growth of choriocarcinoma cells transplanted into nude mice in vivo [9,71,88]. As demonstrated, blockage of hyperglycosylated hCG with a specific antibody to hyperglycosylated hCG, or by blocking α- and β-subunit DNA expression, totally blocks choriocarcinoma growth [9,71,88]. These finding all suggest the use of blockage agent such as an antibody to hyperglycosylated hCG in the treatment of choriocarcinoma. Other research indicates that hyperglycosylated hCG, the cytotrophoblast cell invasion promoter in choriocarcinoma, specifically promotes the invasion in implantation of pregnancy, and the deep implantation of the villous placental structures that is driven by extravillous cytotrophoblast cells [6,8,87]. These studies used the B152 assay for hyperglycosylated hCG. Laboratory experiments show that antibody to hyperglycosylated hCG, antibody B152, blocks growth of cytotrophoblast cell lines in vitro (JEG-3 and Jar cell line). Hyperglycosylated hCG promotes growth of cytotrophoblast cells, at implantation and in choriocarcinoma [9,10,87].

As published [89,90], two third of pregnancy failures, biochemical pregnancies and miscarriages of pregnancy, are due to failure of blastocysts to implant appropriately. The remaining one third of failures are due to hydatidiform mole or genetic abnormalities [89,90]. A total of 62 pregnancies were investigated. On the day of implantation of pregnancy, 42 of 42 term pregnancies produced only hyperglycosylated hCG in vivo (26 of 42 cases) or >50% hyperglycosylated hCG of total hCG. As found, two third of failures (13 of 20) produced insufficient hyperglycosylated hCG or <50% hyperglycosylated hCG of total hCG [8]. It is inferred that pregnancy failures are due to insufficient production of hyperglycosylated hCG leading to failure to implant appropriately. These studies used the B152 assay for hyperglycosylated hCG. Similar finding showing that hyperglycosylated hCG is a marker of pregnancy failure have been reported by Kovalevskaya et al [91], also using the B152 specific assay.

Similarly, hypertense disorders of pregnancy or preeclampsia in pregnancy are due to failure to appropriately connect the implanting villous hemochorial placentation with appropriate uterine blood supply [92,93]. Studies indicate that this may also be due to a deficiency of hyperglycosylated hCG [94].

In conclusion, hyperglycosylated hCG is the invasive signal of cytotrophoblast invasion of pregnancy implantation and choriocarcinoma invasion. Ineffective invasion due to insufficient hyperglycosylated hCG occurs in failed pregnancies, biochemical pregnancies and miscarriages, and seemingly in hypertense disorders of pregnancy.

## Biological function of free β-subunit

The free β-subunit produced is a hyperglycosylated variant of the β-subunit of hCG with triantennary N-linked oligosaccharides and hexasaccharide type O-linked oligosaccharides [95,96]. Excess β-subunit or free β-subunit is produced in hydatidiform mole, choriocarcinoma, and almost exclusively by non-trophoblastic cancers of all primaries.

Studies by Acevedo et al. [96-98], show the presence of hCG free β-subunit in the membranes of all cancer cell lines in vitro, and in all histological samples (slides) of malignancies. This data is considered rather controversial. New data, however, seemingly confirms these findings in cervical cancer cells [99]. Other studies indicate a clear association between detection of free β-subunit in serum samples, or detection of its degradation product, β-subunit core fragment, in urine samples, with cases with poor grade and advanced stage cancer, or poor outcome malignancy [100-103].

In a review of different articles investigating free β-subunit as a prognostic marker in cancer, 12 of 13 studies demonstrated a clear correlation between expression of hCG free β-subunit and poor prognosis [100,104]. These studies together indicate that expression of free β-subunit leads to a negative outcome in human malignancies. Multiple reports now indicate that free β-subunit may have a specific role in malignant transformation of cells [97,99,105-109]. In these, and other studies, stimulation of malignant cell growth has been demonstrated by the action of free β-subunit [97,99,105-109].

Free β-subunit has a major role to play in non-gestational neoplasm biochemistry, either as a promoter causing poor malignancy outcome or as an element involved in malignant transformation. Indeed, efforts are now being directed toward using different hCG β-subunit derivatives as vaccines in the treatment of non-gestational malignancies. Achievement has been reported, with hCG β-subunit immunity improving cancer outcome or cancer survival [110-114]. The association of free β detection and poor prognosis, in combination with site specific hCG β-subunit vaccine technology suggests a plausible route to the development of adjuvant cancer therapies specifically targeting patients with free β-subunit producing non-gestational tumors.

Both hyperglycosylated hCG and free β promote cancer cell growth and malignancy [5,9,87,100,104,107,108], similarly, both hyperglycosylated hCG and free β function by blocking or antagonizing apoptosis causing cell growth [71,99,100,104,107,115]. In the action of both hyperglycosylated hCG and free β the use of the TGFβ receptor is indicated [107,108,116-121]. As reported, free β is produced by bladder cancer cells and inhibits TGFβ activity in bladder cancer cells [122]. Free β-subunit antagonizes TGFβ functions in bladder cancer cells leading to growth and malignancy [33,122]. It is inferred that both hyperglycosylated hCG and hyperglycosylated hCG free β function similarly, both promoting cell growth, invasion and malignancy by blocking apoptosis through antagonizing a TGFβ receptor. We hypothesize that they both bind the same receptor and function through similar pathways.

## Biological function of pituitary hCG

Publications in the nineteen sixties, seventies and eighties suggested bacteria, crabs, and other bazaar sources to explain detection of hCG in non-pregnant individuals, including cancer cases [123-125]. We now have a better understanding of the various not pregnant sources of hCG. Possibilities today include non-trophoblastic cancer, as described in the preceding section, quiescent gestational trophoblastic disease [126], familial hCG syndrome [127], and as shown in this chapter pituitary hCG.

It is now been 30 years since hCG was first demonstrated to originate from the pituitary gland [128]. Since then, almost 40 publications have confirmed this observation and described how very low level hCG production accompanies luteinizing hormone (LH) production at the time of the mid-menstrual cycle pre-ovulatory surge, a normal part of normal pituitary physiology [128-134]. Most noticeably, pituitary derived hCG is normally elevated along with pituitary LH and FSH in women receiving an oophorectomy, during oligomenorrhea in perimenopause (age 40-50), and during amenorrhea in menopause (Age >50) [129-134]. In medical practice, a positive hCG test prior to menopause suggests pregnancy or gestational trophoblastic disease [132-134]. A positive hCG test in perimenopausal and menopause, can represent a predicament to physicians. When an hCG positive patient is referred to an oncologist, they may be considered as having gestational trophoblastic diseases or a non-gestational malignancy, and may be placed on chemotherapy or given hysterectomy with the hope that hCG will disappear. The hCG level will not change in patients treated this way, since it is natural hormone, pituitary hCG.

Pituitary hCG has an identical amino acid structure to pregnancy hCG. It is unique, however, in having a variable portion of sulfated oligosaccharides [133]. The sulfated groups are attached to N-acetylgalactosamine residues, which replaces galactose and sialic acid residues in N- and O-linked oligosaccharides. As found pituitary hCG with sulfated oligosaccharides has a shorter circulating half life that pregnancy hCG [133].

As shown by Odell and Griffin [135,136], using an ultra-sensitive radioimmunoassay LH (sensitivity 0.005 mIU/ml), pituitary hCG is produced at very low levels (mean 0.01 mIU/ml) in men, with a wide range of 0.03 to 1.7 mIU/ml. Pituitary hCG was detected in women in pulses in the luteal and follicular phases of the menstrual cycle which paralleled LH levels [135,136]. Injections of Gonadotropin releasing hormone (GnRH) were shown to directly promote circulating pituitary hCG levels in men and women, just as they similarly promotes LH levels [135,136]. It is inferred that pituitary hCG supplements pituitary LH in men and women [13,136]. The USA hCG Reference Service recently examined over 8300 urine samples from women with normal menstrual periods using the Siemens Immulite 1000 assay to total hCG [13,136]. As found, hCG (sensitivity >1 mIU/ml) was detected at the time of the mid-cycle LH peak in 232 of 277 (84%) menstrual cycles. The mean hCG level was 1.54 ± 0.90 mIU/ml and the range was <1 to 9.2 mIU/ml. This recent study very much confirms the findings of Odell and Griffin showing that pituitary hCG mirrors pituitary LH levels [135,136].

The mass of hCG stored in an individual human pituitary gland, 0.5-1.1 μg hCG per gland, is approximately 25-50 fold less than the mass of LH [146]. Publications show that pituitary hCG has approximately half the biological activity in promoting progesterone production of placental hCG [133]. As such, it is 40-fold more potent than pituitary LH [15]. As indicated by Odell and Griffin, circulating levels of hCG during the menstrual cycle are approximately 1/120th of circulating levels of LH, mIU/ml to mIU/ml [135,136]. Considering the 40-fold greater potency of pituitary hCG, pituitary hCG may therefore have an average potency of approximately 1/3rd of the potency of LH. This makes pituitary hCG a significant pituitary hormone.

**Figure 1:**
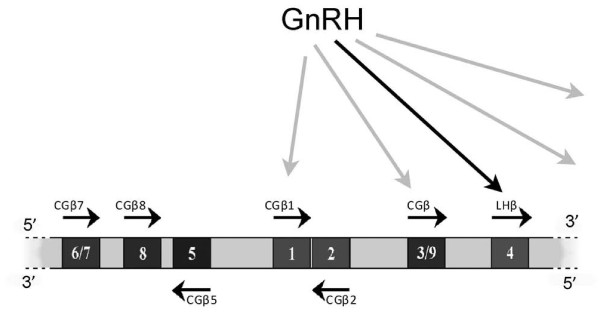
**A diagrammatic representation of the arrangement of genes in the LHβ/hCGβ gene cluster on chromosome 19q13.32**. The gray arrows show the postulated chance stimulation of hCG β-subunit genes by the GnRH promoting LH β-subunit gene transcription.

As yet, it is unknown whether there is a specific function for pituitary hCG during the menstrual cycle. Pituitary hCG could have functions separate from those of LH. But even if pituitary hCG has no specific function, there is a natural explanation for its production. There is a single LH β-subunit gene next to the 8 back-to-back hCG β-subunit genes on human chromosome 19 (Figure 1) [137]. hCG and LH share a single common α-subunit. It is possible, as indicated in Figure 1, that hCG β-subunit gene transcription is promoted consequentially by gonadotropin releasing hormone (GnRH) alongside specific LH β-subunit stimulation in pituitary gonadotrope cells during normal menstrual cycle physiology in women and normal physiology in men.

LH has multiple functions during the LH peak and ovulation period. We know that both LH and hCG act on the hCG/LH receptor to promote progesterone production by corpus luteum cells [17-23]. We assume that LH and hCG act on the same receptor on granulosa and theca cells. As established, with the appearance of an hCG/LH receptor on granulosa cells of the Graafian follicle or primary follicle, LH first promotes follicular growth [138,139], then stimulates diploid cell meiosis [140,141]. LH causes the follicle to form a stigma or protrusion [142], and then promotes collagenase production to degrade and penetrate the stigma [142,143]. The penetration of the stigma causes bursting of the follicle (ovulation) to occur. LH then acts to differentiate the burst or ovulated follicle into a corpus luteum [144,145]. It is not clear whether hCG just incidentally assists LH in each of these steps or has specific functions of its own in one or more of these steps.

## hCG and hyperglycosylated hCG variation

hCG and associated molecules seeming have a wide array of biological activities. hCG and hyperglycosylated hCG effective control placentation and fetal development during pregnancy. Considering these critical biological functions there is a major paradox that exists. That is that individual hCG levels in serum (Table 2) and urine (Table 3) of total hCG and hyperglycosylated hCG vary extremely widely. In serum, in the 4^th ^week of gestation (weeks following start of menstrual period), individual total hCG values vary by 824-fold, between 0.21 and 173 ng/ml amongst different women with singleton term outcome pregnancies (all failing pregnancies removed from table) (Table 2). Hyperglycosylated hCG values vary even wider during this week of pregnancy, 888-fold. In the 5^th ^week of gestation total hCG values vary by 704 fold, between 1.86 and 1308 ng/ml amongst different women with singleton term outcome pregnancies. Hyperglycosylated hCG values vary once again slightly wider, 734-fold. We ask how and why does this extremely wide variation exist with such important molecules between different pregnancies, and how with such extreme variation in signal, can all these pregnancies go to term and produce similar size babies? This is the subject of two articles. The first article addresses the cause of the wide variation [147], and the second article address the affect of the wide variation, or how the receptors cope with this situation [Cole LA, paper submitted to *J Clin Endocrinol Metab*].

**Table 3:** Concentration of total hCG and in 4246 urine samples from 574 women having term pregnancies measured using the Siemens Immulite 1000 total hCG assay.

Gestation age (weeks since start of menstrual period)	N	Median Total hCG ng/ml	Range Total hCG ng/ml (variation)	Variance
3-weeks-3-weeks 6-days	n = 574	0.24 (255 of 574 <0.1 ng/ml)	0 - 415	
4 weeks-4 weeks 6-days	n = 574	21.7 (20 of 574 <0.1 ng/ml)	0 - 213	
5 weeks-5-weeks 6-days	n = 574	301.2	2.3 - 4,195	1839X
6-weeks-6-weeks 6-days	n = 574	1,472	14.1 - 24,580	1743X
7 weeks-7 weeks 6-days	n = 574	4,795	93.1 - 28,370	305X
8 weeks-8 weeks 6-days	n = 574	6,813	124.5 - 42,120	338X
9 weeks-9 weeks 6-days	n = 65	8,869	134.5 - 54,530	405X
10 weeks-10 weeks 6-days	n = 45	9,864	123.4 - 60,130	487X
11 weeks-13-weeks 6-days	n = 74	1,984	179.3 - 49,540	276X
14 weeks-17 weeks 6-days	n = 494	768.8	58.5 - 8,411	143X
18 weeks-26-weeks 6-days	n = 74	506.3	84.0 - 2,643	31.5X
27 weeks-40 weeks 6-days	n = 50	522.4	66.1 - 1,873	28.3X

Data from a further 97 pregnancies that failed (miscarriage) were excluded from this table. Pregnancies which failed to implant in early pregnancy (total hCG <0.1 ng/ml) are indicated in parenthesis.

As published [147], pregnancy data in normally anchored to the start of the last menstrual period. Implantation of term outcome pregnancy (biochemical pregnancies and miscarried pregnancies excluded) or the start of gestation occured, however, anywhere from day 16 to day 32 from this anchoring point in 82 woman with menstrual cycle of average length of 27.7 ± 2.4 days [147,148]. In a 28 day cycle this can be considered as anywhere from 12 days prior to missing a menstrual period to 2 days after the time of missing a menstrual period. The day of implantation is 3 to 16 days after the LH peak. The day of implantation is the day of starting viable pregnancy. Dating this important date to the time of the start of the last menses (weeks of gestation) is a source of great variability [147,148]. If pregnancies were dated, however, to the day of implantation (difficult to measure and difficult as an anchor date), variation is dropped significantly [147]. Normally, at the 4^th ^week (28 days) since the last menstrual period urine hCG ranges from <0.1 ng/ml to 213 ng/ml (Table 2). This suggests variation of >2130-fold. If the same pregnancies were dated to 7 days after the time of implantation, hCG ranges from just 3.1 ng/ml to 402 ng/ml indicating a variation of just 131-fold, a significant difference, p = 0.00005. Clearly dating pregnancies to time of implantation is preferable to dating to start of last menstrual period, but is difficult. Difficult in that it requires daily hCG measurements while attempting to achieve pregnancy to determine time of implantation. Clearly dating of pregnancies is a cause of variation.

Examining 594 pregnancies from implantation to term, only one other cause could be found for individual variations in hCG. That is hCG daily increase rate in the first 4 weeks following implantation. hCG daily increase rate was measured in 82 women (all results from women with biochemical or miscarried pregnancies removed) with term outcome pregnancies, from day of implantation for 28 days. The increase rate per day was averaged over the 28 days. The increase rate per day ranged from 1.52-fold per day to 2.92-fold per day among the 82 women [147]. If this is considered over 7 days then it is equivalent to 1.52^7 ^and 2.92^7 ^or to an increase of 18.7-fold vs. an increase of 1810-fold. Individual hCG daily amplification rate is also a major cause of variation.

It is a fact that pregnancy to pregnancy hCG levels vary greatly. How does the human body deal with these wide variations in hCG concentrations? How does each woman end up with a normal term delivery? This was carefully investigated [Cole LA, paper submitted to *J Clin Endocrinol Metab*]. Most hCG and hyperglycosylated hCG-related parameters, like promotion of uterine vascular angiogenesis, promotion of implantation, differentiation of cytotrophoblast cells, growth and differentiation of fetal organs, are not readily measurable in normal term pregnancies. One hCG-related biological activity was, however, measurable, promotion of progesterone production by corpus luteal cells at 3-6 weeks of gestation. Serum progesterone was measured during the 4^th ^week of gestation, in those providing serum samples.

During the 4^th ^week of pregnancy serum hCG ranged widely from 0.21 to 173 ng/ml or variation of 824-fold. Serum progesterone during this same period, in these same women, did no vary widely, 6.5 to 101 ng/ml, or 16-fold (paper submitted to *J Clin Endocrinol Metab*). The median progesterone concentration was 22 ng/ml. Interestingly, in the case with extremely low serum hCG concentration, 0.21 ng/ml or 23 mIU/ml, the progesterone was slightly higher than the median, 36.1 ng/ml. In the case with extremely high hCG concentration, 173 ng/ml or 1903 mIU/ml, the progesterone concentration was 11.4 ng/ml, or slightly lower that the median. The hCG levels stretched 824-fold from 0.21 to 173 ng/ml, but the resulting progesterone concentrations or biological activity stretched just 3.16-fold from 11.4 to 36.1 ng/ml, why we ask?

This is apparently due to the hCG/LH receptor spare receptor concept [149-151]. Under the spare receptor concept, when only a tiny proportion of receptors is activated in a cell it may yield a similar cellular response to all receptors on the cell being activated [149-151]. This is the best explanation of these findings. Similarly, in cases with extremely high serum hCG concentration lower than normal progesterone was observed. This is seemingly due to receptor down-regulation in the presence of high concentrations of hCG [152-154]. As demonstrated, high concentrations of hCG decrease the number of receptor on cells by degrading the receptor transcript in cells reducing their half-life [152]. Just at the spare receptor mechanism deals with low hCG concentrations at the receptor level, the down-regulation mechanism deals with high hCG concentrations at the receptor level. Assumingly the same as reported to happen at the corpus luteal hCG/LH receptor occurs at the decidua, myometrium, fetal organ, uterine vasculature and human brain receptors. Assumingly, it also happens at the hyperglycosylated hCG or TGFβ antagonism site. The end result is that the hCG and hyperglycosylated hCG variation paradox may be a simple case of "nature takes care of it."

## The hCG/LH receptor

The hCG/LH receptor is located on corpus luteal cells of the ovary for promotion of progesterone, on the decidua for initial communication with the blastocyst on myometrial tissue for growth in line with fetus and for muscle relaxation, on uterine vasculature for angiogenesis, on umbilical cord tissue for growth, on fetal organs for growth and differentiation, on cytotrophoblast cells for differentiation, and on human brain cells, leading to hyperemesis gravidarum. The hCG/LH receptor responds to hCG, LH and hyperglycosylated hCG, but not hCG free subunits or nicked hCG [155]. There is solid evidence showing that the α-subunit has a role in receptor binding and β-subunit has a function in receptor specificity [156,157]. The human hCG/LH receptor comprises 675 amino acids [158-160]. The hCG/LH receptor is located on human chromosome 2p21, with 11 exons and 10 introns [161-163], exons 1-10 and a portion of exon 11 encode the extracellular domain [164]. hCG/LH receptor sequence and cloning studies indicate that it is part of a large family of guanine nucleotide binding proteins (G-protein), membrane coupled receptors [165,166].

**Figure 2:**
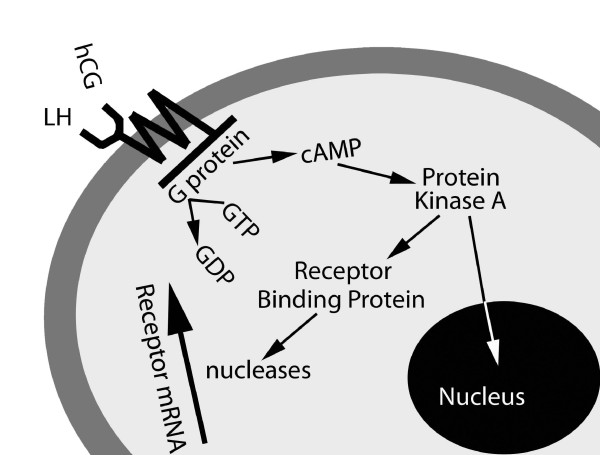
**Activation of hCG/LH receptor, G protein and cAMP, protein kinase expression, and production of hCG/LH receptor binding protein (LHRBP)**. Synthesis of LHRBP activates exo- and endonucleases which destroy receptor mRNA, limiting expression and down regulating the receptor.

Stimulation by hCG or LH, activates the G-protein, resulting in the stimulation of the membrane bound adenylate cyclase (Figure 2). Activation of adenylate cyclase catalyses the conversion of ATP to cAMP elevating intracellular levels (Figure 2). Following upregulation of cAMP, activation of phosphokinase A then occurs, resulting in phosphorylation and activation of the cAMP responsive element [159].

Activation of protein kinase activates the mitogen protein kinase pathways and a Janus-kinase signaling pathway [167]. All endocrine functions involve DNA transcription or generation of mRNA. Promotion of progesterone production in corpus luteal cells involves the synthesis of cholesterol side-chain cleavage enzyme. Fetal tissue growth involves protein synthesis. A parallel mechanism enhances the synthesis of an hCG/LH receptor binding protein. This activates exo- and endonuclease and leads to the destruction of receptor mRNA. This mechanism limits receptor expression, effectively down-regulating the receptor [168].

Other studies indicate an inositol phospholipid protein kinase-C mechanism is involved in the action of hCG/LH receptors [159]. Davis and colleagues [169] and Guderman and colleagues [169] show that LH and hCG stimulate a phospholipase C, leading to stimulation of protein kinase C and activation of hCG/LH receptor. Moreover, recent data suggest that in the case of hCG signaling at implantation and production of natural killer cells binding with a mannose receptor may be the activation mechanism [170,171]. Mutiple pathways are proposed here for hCG/LH receptor activation. While they may all be effective in different cells, they all have to be carefully considered as parts of the principal receptor mechanism.

## Competing interests

The author declares that they have no competing interests.
